# Zeolite Clinoptilolite: Therapeutic Virtues of an Ancient Mineral

**DOI:** 10.3390/molecules24081517

**Published:** 2019-04-17

**Authors:** Andrea Mastinu, Amit Kumar, Giuseppina Maccarinelli, Sara Anna Bonini, Marika Premoli, Francesca Aria, Alessandra Gianoncelli, Maurizio Memo

**Affiliations:** 1 Department of Molecular and Translational Medicine, Division of Pharmacology, University of Brescia, 25123 Brescia, Italy; giuseppina.maccarinelli@unibs.it (G.M.); sara.bonini@unibs.it (S.A.B.); m.premoli002@unibs.it (M.P.); f.aria@unibs.it (F.A.); alessandra.gianoncelli@unibs.it (A.G.); maurizio.memo@unibs.it (M.M.); 2 Department of Neurobiology, Care Sciences and Society, Center for Alzheimer Research, Division of Clinical Geriatrics, Karolinska Institutet, 14183 Huddinge, Sweden; amit.kumar@ki.se

**Keywords:** zeolite clinoptilolite, micronization, tribomechanical activation, detoxifying, antioxidant, anti-inflammatory, Alzheimer disease

## Abstract

Zeolites are porous minerals with high absorbency and ion-exchange capacity. Their molecular structure is a dense network of AlO_4_ and SiO_4_ that generates cavities where water and other polar molecules or ions are inserted/exchanged. Even though there are several synthetic or natural occurring species of zeolites, the most widespread and studied is the naturally occurring zeolite clinoptilolite (ZC). ZC is an excellent detoxifying, antioxidant and anti-inflammatory agent. As a result, it is been used in many industrial applications ranging from environmental remediation to oral applications/supplementation in vivo in humans as food supplements or medical devices. Moreover, the modification as micronization of ZC (M-ZC) or tribomechanically activated zeolite clinoptilolite (TMAZ) or furthermore as double tribomechanically activated zeolite clinoptilolite (PMA-ZC) allows improving its benefits in preclinical and clinical models. Despite its extensive use, many underlying action mechanisms of ZC in its natural or modified forms are still unclear, especially in humans. The main aim of this review is to shed light on the geochemical aspects and therapeutic potentials of ZC with a vision of endorsing further preclinical and clinical research on zeolites, in specific on the ZC and its modified forms as a potential agent for promoting human brain health and overall well-being.

## 1. Introduction

Zeolites represent a family of hundreds of microporous mineral members known for their ion-exchange properties [1]. The term “zeolite” coined in 1756 by the Baron Axel Fredrik Cronstedt, a Swedish mineralogist and chemist, derives from the Greek language ζέω (“zeo”, to boil) and λίθος (“lithos”, stone) meaning “boiling stone”. The structure of the zeolites mainly consists of alumino-silicates with SiO_4_ and AlO_4_ structures connected by shared oxygen atoms [2]. Clinoptilolite is one of the most abundant natural zeolites, widely distributed throughout the world and used for its ion exchange and adsorbent properties [3]. Thanks to its remarkable and unique ion-exchange properties in water, it has been employed for various medical, industrial and environmental uses, in particular, for sequestration of toxic pollutants from industrial effluent and wastes [3]. At present, many positive effects are recognized due to the capacity of natural occurring zeolite clinoptilolite (ZC) to adsorb and therefore remove harmful substances like heavy metals, ammonia, or other small molecules in the gastrointestinal tract of humans. It is important to mention that this positive impact can improve through modifications of ZC due to micronization of ZC (M-ZC) or tribomechanical activation of ZC (TMAZ) or double tribomechanical activation of ZC (PMA-ZC), known as Panaceo Micro Activation [4]. This recently developed PMA (Panaceo Micro Activation)-technology is a special tribomechanical micronization process that causes self-collisions of zeolite particles and contributes to increasing the surface charge and the cation exchange of the individual particles, improving the overall therapeutic performance of ZC [5]. The oral application of the specific PMA-zeolite in a randomized clinical trial in humans furthermore suggests having a positive impact on the intestinal tract as it positively influenced the intestinal wall integrity [6]. Preclinical data furthermore suggest a positive impact on the intestinal microbiome [7]. This connection might be interesting as recent studies have established a possible link between the gut microbiome and chronic neurological disorders. This possible link has been termed as the “Gut-Brain Axis” [8].

In order to gain a better understanding of natural zeolite minerals and their therapeutic action and some side effects, it is essential to study their geological origin, chemical composition, and structure. This review will focus on exploring the geo-chemical aspects of naturally occurring zeolite clinoptilolite (ZC) without any modification—referred to either as ZC or “natural zeolite” or “zeolite” in this article. Furthermore, the focus will be on the modified forms of ZC (M-ZC, TMAZ, PMA) by highlighting its therapeutic aspects and with the aim of promoting further research on ZC for overall human well-being and as a potential preventive agent in Alzheimer disease and other neurological disorders.

## 2. Where Do Zeolites Come From?

**Figure 1:**
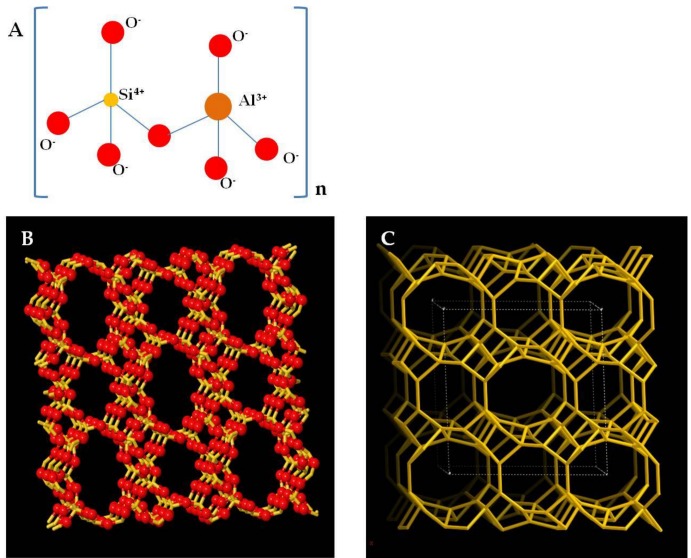
(**A**) AlO_4_ and SiO_4_ tetrahedrons joined by oxygen atoms that constitute the reticular structure of the zeolite. (**B**) and (**C**) Clinoptilolite framework type by http://www.iza-structure.org/.

The geological formation of natural zeolite indifference to synthetic zeolite involves different rock species, in particular, physio-chemical conditions of temperature and pressure. These rock species could vary from volcanic to plutonic rocks [9,10]. Below the surface of the earth, magma releases heat, modifying the structure of plutons previously solidified in specific contact areas known as aureoles. In these aureoles, different porous zeolite mineral formations take place upon deposition of water, for example, as in the porous tephra, which is produced by viscous magma with a high content of silicon, or as in the volcaniclastic sediments or in the feldspathic sandstone [9]. In nature, the genesis of several zeolite species depends on the type of minerals present (for example, an acid or basic volcanic glass) which characterize its structure. Zeolite rocks are particularly widespread throughout the earth’s crust, particularly in the Cenozoic and Mesozoic layers and clinoptilolite is considered as the most common naturally occurring zeolite mineral [11,12,13,14,15]. Zeolites are distributed mainly in regions with a past volcanic history or areas still geologically active today [11]. Indeed, stratigraphy belonging to different geological eras ranging from Tanzawa Mountains (Japan) to Yellowstone Park (USA), from Tecopa Lake Beds in Southern California to the Eocene Wagon Bed Formation in Wyoming, from Paleogene and Cretaceous calcareous sediments in the Atlantic and Pacific Ocean to bituminous sediments in dolomitic shale, are rich in natural zeolite-clinoptilolite [16]. On the other hand, hydrothermal zeolites are mainly found in the newer orogenic belts and hot spots where heavy deposition and recent volcanic events occurred, such as in the Tethys regions including France, Italy, Switzerland and Slovenia [17,18]. Moreover, the process of zeolite formation from feldspathic rocks called “zeolitization” is still ongoing in active volcanic areas and even today we can witness these zeolitic transformations in volcanic regions with thermal water sites [19,20]. From a chemical point of view, zeolites show certain chemical stability in aqueous solutions at different pH values [20]. The clinoptilolite structure shows high thermal stability which is essential for human applications. It is still not destroyed after heating 12 h at 750 °C [21]. The presence of hydrogen and hydroxyl ions in solution generate physical-chemical phenomena such as hydrolysis, degradation, dissolution and phase transformation which help in the characterization of different types of mineral zeolites. The zeolites have a microporous network composition consisting of tetrahedral of AlO_4_ and SiO_4_ joined by shared oxygen atoms [22] (see Figure 1).

**Figure 2:**
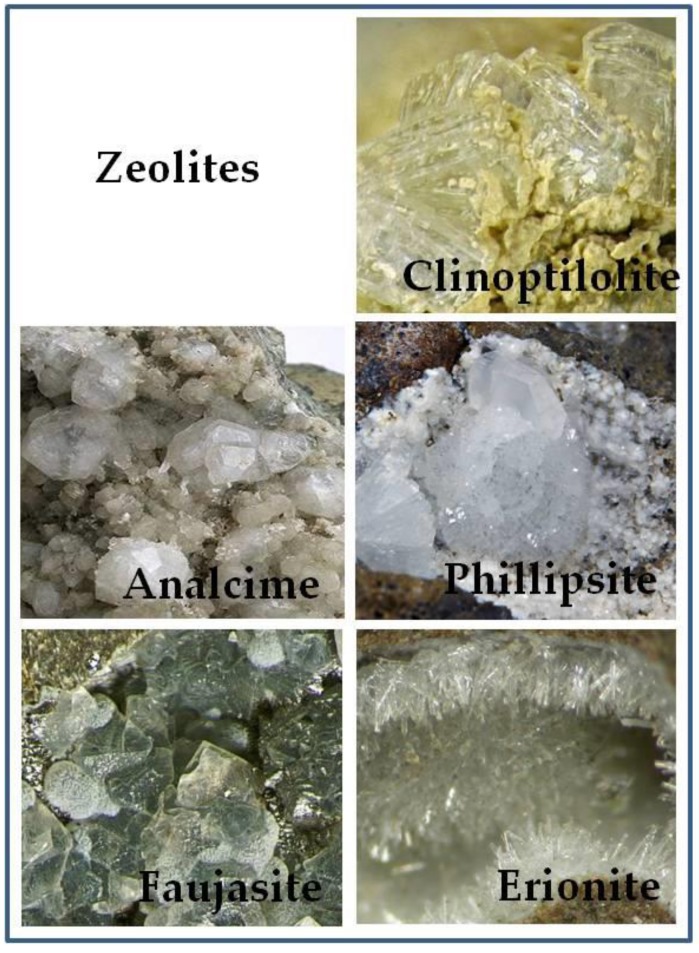
Some minerals with zeolite structure. Clinoptilolite photo by Christian Rewitzer Collection (CC BY-SA 3.0); Analcime photo by Carles Milan (CC BY 3.0); Phillipsite photo by Martin Zinn (CC-BY-SA-3.0); Faujasite photo by Modris Baum (Public Domain); Erionite photo by Leon Hupperichs (CC BY-SA 3.0).

In nature, there are more than 80 different types of zeolites, whose particles can be grouped in crystalline structure with a fibrous, lamellar or spheroid morphology [23]. These types of zeolite (Figure 2) show different characteristics and applications summarized in Table 1. The most widely used and diffused zeolite is ZC. The whole structure of ZC is made of pores or channels with a uniform shape in which the substances can be adsorbed. In particular, water, or polar molecules, are inserted into these pores that act as sieves for molecules that interact with zeolites by dipolar interactions [22]. In addition to water, the cavities and channels are also occupied by monovalent alkaline ions, such as sodium and potassium, and divalent atoms such as calcium and barium, or hydroxyl groups that can easily be exchanged with other polar molecules and cations from the surrounding environment [24]. The zeolites ion exchange capacity and cationic exchange within its pores/channels mainly depend on its silicon and aluminum ratio [24]. This ratio is furthermore important for the stability of ZC. It has been documented that zeolites with higher silica content, such as clinoptilolite, are stable under acid conditions [25]. In vivo experiments with TMAZ and PMA-ZC proved stability in particular to possible aluminum ions leakage into the body under the conditions of the gastrointestinal tract. These experiments confirm the safety as no increased aluminum ions concentrations were detected neither in serum nor in organs of animals after supplementation with PMA-ZC and TMAZ [4]. The pores of the ZC, in addition to metal cations and water, can interact with other molecules and ionic groups of organic origin, such as ammonia and nitrates, which bind with different levels of affinity [26,27]. Thanks to these exceptional ion interactions properties, ZC is considered as one of the key important inorganic cation exchangers and heavily applied in many industrial sectors such as in the treatment of waste-water, nuclear waste, cultivated fields, animal husbandry, or as oral supplementation in humans [5]. The ability to interchange ions leaves open some questions about the selectivity of ZC. Indeed, the impossibility of ZC to absorb useful ions for health still needs to be clearly demonstrated.

Moreover, not all types of zeolite are safe. In particular, it has been found that zeolite erionite is associated with lung cancer and malignant mesothelioma [28,29]. In particular, erionite forms fragile wool-like fibrous masses in the cavities of rock formations [10] and shows properties similar to those of asbestos as malignant mesothelioma development in humans [30]. Mesothelioma is a tumor that originates from the mesothelial cells that line the pleural, pericardial and peritoneal surfaces. Some studies have reported that continued exposure to erionite fiber dust has been the cause of deaths in some villages in Turkey [31]. These important side effects of zeolite have been abundantly documented only for the erionite type. Other fibrous zeolites such as offretite and scolecite can alter some mitochondrial mechanisms and the organization of the cellular structure [32]. This review has focused its attention on ZC, a natural zeolite which showed no toxic effects but potential therapeutic applications. Despite the absence of toxicity of ZC, some authors have highlighted potential side effects that must be considered when using ZC [5,33]. In particular, ZC reduced cell viability and DNA synthesis and increased cell apoptosis in in vitro models [34]. ZC has reduced the functionality of key proteins involved in cell survival and apoptosis in tumor cell lines. This action on tumor cell lines has been attributed to the absorptive and ion-exchange capacities of ZC that can modify the concentration of calcium ions and affect the Ca-dependent molecular signals. The main characteristic of ion exchanger of the ZC can represent a criticality that needs further study to evaluate all the benefits of this food supplement.

## 3. Zeolite Clinoptilolite: A Multifaceted Rock

ZC is the zeolite most used and studied in medicine and in particular, the ZC tribomechanically micronized one (TMAZ) or two times (PMA) with PMA technology (PMA-ZC) showed some preclinical and clinical evidences [4,5,44,45]. Several studies conducted in the last decades have shown high efficacy of ZC in various medical applications both in vitro and in vivo. Moreover, different processing procedures are responsible for substantial changes in the physical–chemical properties like absorbent characteristics or ion-exchange capacities or the surface area of ZC. Those can be improved thanks to particular treatments such as micronization or the particular tribomechanical micronization. For human applications, it is important to use ZC which is considered safe for oral applications in humans. Recently published data suggest that the PMA-ZC show improved physical–chemical properties might be a safe option [5]. This improved physical–chemical properties seem to be the basis of its therapeutic virtues.

In this section, the characteristics of different modified ZCs and their therapeutic applications will be discussed.

### 3.1. Micronized Zeolite Clinoptilolite: A Special Treatment

The micronization of zeolite clinoptilolite (M-ZC) is a procedure that breaks down the ZC into small parts accentuating its detoxifying characteristics [44]. This micronization occurs with mills having two counter-rotating parallel blades, which, with a thrust of 2500 km/h and particular aerodynamics, create two counter-rotating airflows [46]. This ZC powder is distributed in the two airflows and begins to self-collide with a frequency of 3–4000 collisions/s [46]. Furthermore, tribomechanical micronization is a specific micronization first used to activate ZC one time—TMAZ and was further developed as Panaceo Micro Activation technology with PMA-ZC as a result [6]. Thus obtained, it underwent a very strong ionic excitement, which increased its surface charge and detoxifying capacity towards toxins, radicals, ammonia, and heavy metals [5]. However, the main element is given by the structural modification making the PMA-ZC much more active in the “recall” in the intestinal lumen. In fact today, PMA-ZC is prescribed for absorbing/chelating and removing harmful and toxic substances from the gastrointestinal tract (e.g., heavy metals, nitrosamines, ammonia, mycotoxins, cations, radioactive materials, pesticides), reducing their absorption in the body [4,6]. In addition, a clinical study shows that it strengthens the intestinal wall which might be based on its absorbing properties [6]. Finally, it can also function as an anti-oxidant by capturing free radicals and reducing the formation of reactive oxygen species, as discussed below.

### 3.2. Detoxifying Effects

**Figure 3:**
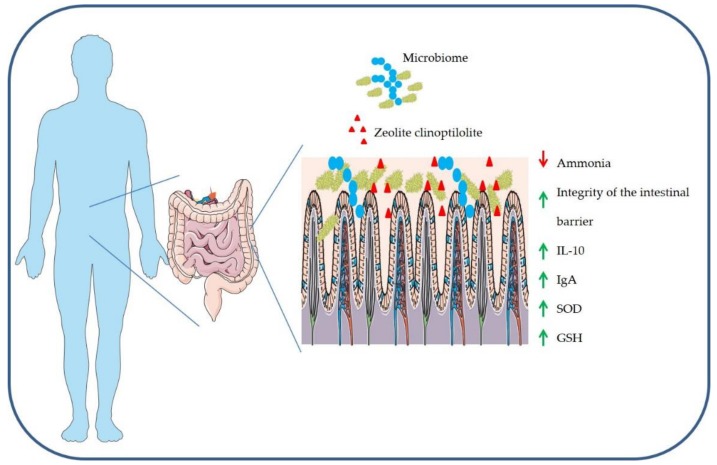
Zeolite clinoptilolite (ZC) reduces ammonia concentration and improves the integrity of the intestinal barrier. Furthermore, ZC acts on intestinal lymphoid tissues with a positive impact on the intestinal ecosystem and boosting the immune system. ZC avails metal ions, present within its structure, as cofactors for the activation of antioxidant enzymes.

Most of the clinical positive effects of ZC and modified ZC have been attributed to its reversible ion exchange and adsorption capacity. The first detoxifying effects of ZC in a modified form were observed in murine models. In lead poisoned mice, the modified ZC reduced the accumulation of lead in the intestine by more than 70% with a protective effect on the brain tissue [47]. In rats intoxicated with organophosphates, ZC was effective in restoring cholinesterase activity at the system level [48,49]. According to some reports, ZC in the intestine could bind to the organophosphate through an interaction with an -OH group or a dipole–dipole interaction and therefore can be excreted in the feces [50]. As a result, the role of ZC has been recognized in zootechnics and veterinary medicine where it has provided clear evidence on improving the physical fitness and efficiency of farm animals by removing numerous harmful substances from the body including ammonia, nitrates, mycotoxins, and other toxins [51]. The presence of ammonia and polar molecules in the environment is the result of intensive zootechnical and agricultural activities that pour these pollutants into the environment, contaminating our groundwater resources. ZC has shown a high ability to eliminate ammonia from the environment, especially from drinking water [3]. Moreover, today it has also been used in the reclamation of animal manure before soil contamination [52]. In addition, dairy cattle may undergo nitrate and mycotoxins intoxication from the water, which can generate alterations in protein and glucose metabolism [53]. In these cows, the integration of ZC in the feed has reduced the assimilation of nitrates and concentration of aflatoxins in their milk, improving the systemic toxic effects [53,54]. The detoxifying action on these ions has no effect on the physiological ionic equilibrium. In fact, the blood mineral levels of cattle were unaffected by integration with ZC [55]. In addition, similar beneficial effects have also been observed in other farm animals such as pigs and poultry. In this regard, ZC has shown a positive effect in farmed poultry in balancing the total intestinal microbial flora, reducing toxic effects of aflatoxins and increasing the antioxidant activity (see below) of peroxidase, catalase, and SOD (superoxide dismutase) as well as increasing levels of omega-3 fatty acids in eggs [56,57,58]. The physical–chemical properties and therefore detoxifying abilities improve when ZC is subjected to PMA technology as observed in some clinical settings. In particular, the detoxifying action of PMA-ZC towards ammonia may have potential applications as a therapeutic adjuvant in humans as hypothesized based on results from a clinical study [5,6]. Ammonia is produced as a waste in the body during the metabolism of proteins, transformed by the liver into urea, and eliminated by kidneys [59]. Diets rich in proteins, pathologies with excessive protein fermentation, as in the case of irritable bowel and ulcerative colitis lead to an increase in the production of ammonia [59]. High levels of ammonia indicate poor hepatic and renal function. Some authors have highlighted the important ammonia detoxifying contribution of ZC in various diseases [60]. In particular, a study focused on the administration of specific PMA-ZC (Panaceo Sport) to endurance-trained subjects who probably undergo a rich protein diet and frequently encounter intestinal symptoms such as nausea, stomach, and intestinal cramps, vomiting, and diarrhea [6]. These annoying conditions can result from the excessive protein fermentation accompanied by the higher release of ammonia in the intestine. Excessive exercise can also compromise the same intestinal permeability and trigger cell-mediated inflammatory responses [61]. Supplementation with PMA-ZC improved the integrity of the intestinal barrier, decreasing the concentrations of zonulin, a marker of increased intestinal permeability and might furthermore have positive effects on nausea and diarrhea (Figure 3) [6]. Despite these pieces of evidence on the detoxifying action of ZC, there are still few clinical studies in humans. The ability to interchange ions of ZC raises some questions regarding the risk of altering the homeostasis of necessary trace elements and micronutrients in humans [62]. Indeed, despite studies of the use of PMA-ZC in athletes not revealing any alterations in the hydro-saline equilibrium, further investigations are necessary for human use.

### 3.3. Anti-Inflammatory Effects

Different hypotheses and recently published reports have explained the anti-inflammatory effects of ZC, some of these reports highlighting the relationship between PMA-ZC and the site of action at the intestinal level. The mucosa-associated lymphoid tissue (MALT) and the gut-associated lymphoid tissue (GALT) are the sites where the main intestinal cell-mediated inflammatory responses are modulated [63]. These two tissues are composed of M cells, highly specialized cells, overlying lymphatic nodules called Peyer’s patch [63]. The M cells by endocytosis acquire bacteria, soluble and solid substances through the apical membrane and transport them by means of vesicles to the basolateral membrane where they are released by exocytosis and processed by macrophages. Macrophages present the antigen to T-lymphocytes, which stimulate IgA production [63]. TMAZ immunomodulatory effects may be due to interactions with M cells [64]. Indeed, M cells could interact with ZC particles tribomechanically activated, which could then induce changes in the redox homeostasis and affect Peyer’s patches. These absorbed microparticles do not pass to the bloodstream, but act locally in this district as hypothesized by Lamprecht and colleagues [6]. The action of PMA-ZC on intestinal lymphoid tissues (positive impact on IL-10) was also observed in the metabolites released by probiotics capable of improving the intestinal ecosystem and boosting the immune system [65,66]. Sabbioni and colleagues have observed, in experimental animal models treated with a particular zeolite, chabazitic zeolites, an increase in the production of B-lymphocytes and IgA [67], the main defensive mechanisms of the intestine against pathogenic bacteria [68]. IgA plays a fundamental role in modulating the pro-inflammatory response. Indeed, IgAs balance the body’s immune response to the intestinal microbial flora, helping in the adaptation towards bacteria already present and detecting the presence of new ones [69]. Furthermore, the immunomodulatory action of ZC such as also for TMAZ or PMA-ZC would occur at this level, acting, perhaps, as a superantigen. Superantigens are particular molecules of peptidic or oligo-protein nature capable of directly stimulating a significant and non-specific immune system response [70]. This immuno activation/stimulation happens immediately after the interaction of superantigen with the class II MHC (major histocompatibility complex) molecules and the T-lymphocytes receptors [71]. Then, superantigens activate the T-lymphocytes, which induce the cellular and humoral immune response [70,71]. Even though the stimulation of lymphocytes by zeolites has been demonstrated only in vitro, the mechanism of IgA production after stimulation of T-lymphocytes by ZC can be considered as a valid hypothesis. Finally, other immunomodulatory effects of TMAZ, PMA-ZC, and other zeolites cannot be excluded, but this topic needs further investigation. For instance, a remarkable increase in specific immune cell counts, CD19C B-lymphocytes, CD4C T-lymphocytes, and HLA-DRC activated T-lymphocytes were also observed in patients treated for immunodeficiency and receiving TMAZ, which is similar to PMA-ZC due to the tribomechanical activation with no reports of any significant side effects on the immune system after treatment [64].

### 3.4. Antioxidant Effects

The enzymatic systems that regulate the homeostasis of the redox system in the body are the superoxide dismutase (SOD), catalase, peroxiredoxin, thioredoxin and glutathione (GSH) enzymes [72]. An excess of reactive oxygen species (ROS) produced at the endogenous level by the mitochondria, by cytochrome P450, and by inflammatory cells, or exogenously generated by ionizing radiations, causes damage to DNA, proteins, and lipids [73]. This process is called oxidative stress and is involved in various diseases including obesity, atherosclerosis, neurological diseases, and cancer [74]. The antioxidant capacity of micronized ZC is based on its ability to avail metal ions present within its structure as cofactors for the activation of antioxidant enzymes. In this regard, the most important data were obtained using animal models. In particular, Saribeyoglu and colleagues hypothesized antioxidant properties of ZC towards hepatectomy induced by oxidative stress in rats [75]. Indeed, these rats showed an increase of SOD and GSH activity after ZC treatment. Also, administration of M-ZC and TMAZ reduced hepatic lipid peroxidation in mice treated with doxorubicin [44]. Furthermore, ZC restored the activity of antioxidant enzymes in murine models intoxicated with fluorides [76]. In chickens receiving daily ZC in food, antioxidant capacity was increased by reducing the free radical content of intestinal nitric oxide and increasing the activity of GSH, catalase and SOD enzymes [77,78,79]. Finally, the TMAZ (similar to PMA-ZC) has shown a particular antioxidant action in murine models of Alzheimer’s disease. In particular, the increase in SOD activity also occurred in the brain with a consequent decrease of β-amyloid levels in transgenic mouse models of Alzheimer’s disease after treatment with TMAZ [45]. Despite these results on animal models, still few studies showed a clear effect of all ZC on redox systems in humans. For instance, the integration of PMA-ZC in sports nutrition has no effect on the production of free radicals [6]. In this regard, some studies hypothesize that the antioxidant effect may be a consequence of the detoxifying effects of PMA-ZC at the intestinal and systemic levels which are not always quantifiable [5]. For this reason, further human studies are necessary to understand the effective contribution of all zeolite stones to our redox balance. In particular, further data with already authorized zeolites for oral supplementation (e.g., PMA-ZC) would be useful.

## 4. Brain Effects of Zeolite Clinoptilolite

Given the existence of the gut-brain interconnection, it has been hypothesized that an indirect action of ZC exists at the level of the central nervous system. In this regard, some recent hypotheses are discussed below.

### 4.1. What is the Action of Zeolites in the Brain?

In the pharmacological field, there are so many treatments which have beneficial effects but their underlying molecular mechanisms are still unknown [80]. The same could be true for zeolites. According to Hecht K. and colleagues, ZC is able to reduce stress and improve sleep, producing a soothing effect in humans [81]. To date, the neurological and molecular bases for these soothing effects are still puzzling researchers. Perhaps an answer can come from studies of the murine models. In fact, Mück-Šeler and colleagues have shown that TMAZ can abolish the reduced binding of 3H-8-OH-DPAT to 5-HT1A receptor in cancer mice models and produce possible beneficial effects [82]. Furthermore, a behavioral study showed that the rats react better to environmental stress if their food is supplemented with ZC [83]. As zeolites do not cross the intestinal barrier, and not even the blood–brain barrier when the particles are big enough (no nanoparticles), this suggests an indirect mechanism that acts remotely (gut?) and positively on the brain. For this reason, some authors have hypothesized the possibility of zeolites–in this case, as nano-materials that would be able to cross barriers (intestinal or even blood–brain-barrier) to transport neurotransmitters, peptides, antibiotics, exploiting their capacity of ion exchangers [84,85,86,87]. Unfortunately, this hypothesis is not without difficulties and criticalities. Therefore, these observations on the effects of ZC remain hypotheses for potential applications that will require further in-depth studies.

### 4.2. Zeolites, Gut-Brain Axis and Alzheimer Disease

**Figure 4:**
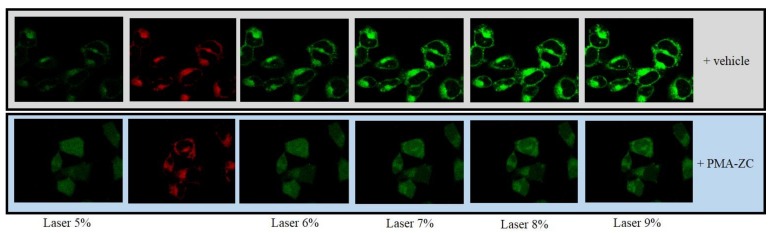
Mitochondrial ROS production in SH-SY5Y neuronal cells. In this particular set of images, a set of cells (lower panels) were preincubated with tribomechanically activated zeolite (TMAZ) and Panaceo Micro Activation (PMA-ZC) (2,5 ng/mL) or vehicle (upper panels) for 30 min before the laser excitation. Cells pretreated with TMAZ/PMA-ZC were more protected from oxidative stress than cells that received only vehicle (adapted from Montinaro et al., 2013).

Alzheimer disease (AD) is a progressive neurodegenerative disorder and the most prevalent form of dementia affecting millions of people worldwide with no potential cure or treatment in sight [88]. As all the recent clinical trials are failing in final phases, researchers are looking for alternate ways to combat and contrast this deadly disease [89,90,91,92]. One of the ways is to prevent the progression of the disease by promoting a healthy lifestyle through a combination of balanced diet and exercise, as recent studies have established a possible link between the gut microbiome and the chronic neurological disorders [93,94,95,96]. This possible link has been termed as the “Gut-Brain Axis” [8]. Moreover, several studies have shown that the altered or abnormal gut microbiome due to an unhealthy lifestyle could affect intestinal permeability, which may be involved in the progression of AD and several other neurological disorders [97,98,99]. In addition, diets rich in fibers, fruits, and vegetables are more effective in maintaining gut homeostasis/integrity and delaying AD development as compared to a western diet rich in high-fat, red meat and processed food [100]. In this regard, specific authorized ZC, like PMA-ZC which is the further developed TMAZ for human applications, could represent good alternatives in AD patients as they possess detoxifying, antioxidants, anti-inflammatory, and gut-protective properties. A study performed by Montinaro and colleagues, using TMAZ showed protective effects against oxidative stress (Figure 4) and plaque burden in transgenic mice models of AD [45]. They suggested that the protective effect of TMAZ—further developed as PMA-ZC could be due to its ability to maintain gut electrolyte equilibrium. Even though this study showed the extensive potential of TMAZ/PMA-ZC in AD, it is still an unexplored domain and several new and extensive studies (biochemical, preclinical and clinical) are needed to establish its prominent role as a preventive treatment in AD and other neurological disorders.

## 5. Conclusions

The physical–chemical properties of ZC are essential for the absorbing characteristics or ion-exchange, and therefore, for its therapeutic potential. Indeed, the rock mixtures that constitute the natural mineral form complexes of aluminum and silicon which allow the formation of cavities inside where inorganic and organic polar or metal ions are deposited or exchanged. This exceptional ion-exchange capacity, as well as the surface area, are improved with micronization processes, in particular with the tribomechanical micronization processes used for TMAZ or PMA-ZC. In particular, thanks to PMA technology, ZC is activated and its absorbing capacities increase. ZC, in general, is known as an environmental decontaminant and specific TMAZ and PMA-ZC have found multiple applications in the last 20 years. The most important is its oral use for supplementation in humans. Environment decontamination from heavy metals derived from industrial waste or from organic molecules derived from intensive farming is essential for biological ecosystems. Due to the binding capacities, ZC has been generally used in the zootechnical field for water purification and decontamination, contributing to the improvement of livestock health. Furthermore, environmental contaminants accumulate throughout the food chain and are therefore part of foodstuff. For this reason, PMA-ZC is studied and used for oral supplementation to bind toxic substances such as ammonia or heavy metals in the milieu of the gastro-intestinal tract. Furthermore, PMA-ZC is capable of improving the ecosystem of intestinal microbial flora. Based on the data collected on animal models and on a few clinical trials, it can be speculated that the general state of wellbeing generated by ZC, and in particular by TMAZ and PMA-ZC, is due to its detoxifying, anti-inflammatory and antioxidant action in the intestine. Indeed, recent findings of the importance of the gut microbiome in the regulation of immunity and its interconnection with the central nervous system could at least partially explain the results obtained using animal models treated with PMA-ZC. ZC could purify the internal environment of our body, maintain gut microbiota homeostasis for healthy brain activity, improve the antioxidant and endogenous anti-inflammatory activities thereby improving the overall wellbeing of the patient. Despite all this, still, only a few studies have defined the molecular mechanisms underlying the positive effects of all ZC. Furthermore, several important questions are still unanswered: What is the molecular mechanism that increases the antioxidant activity of SOD and GSH in experimental models treated with ZC? What is the translational value of the action of ZC on β-amyloid levels? Is there risk of reducing the contribution of mineral salts due to the ion exchange action of ZC? Finally, is there remote danger of ZC crossing the intestinal wall?

In conclusion, preclinical research on zeolites was not aimed at finding a new drug, but a food supplement that can improve lifestyle and be combined with traditional pharmacological treatment. In fact, recent evidenced suggested a promising detoxifying role of ZC in the removal of toxic metabolites produced by drugs chronically administered during chemotherapy, diabetes, or cardiovascular diseases. However, new and extensive research will be needed to explore all the potential benefits that ZC and other specific modified ZC can produce on human health.

## Figures and Tables

**Table 1:** Characteristics and biological effects of some types of zeolite.

Zeolite Mineral Species	Formula	Effects	Ref.
Analcime	NaAlSi_2_O_6_·H_2_O	Antioxidant	[35]
Phillipsite	(Ca,Na_2_,K_2_)_3_Al_6_Si_10_O_32_·12H_2_O	Antioxidant and gut microbiota	[35,36]
Faujasite	(Na_2_,Ca,Mg)_3.5_[Al_7_Si_17_O_48_]·32(H_2_O)	Antimicrobial	[37]
Erionite	(Na_2_,K_2_,Ca)_2_[Al_4_Si_14_O_36_]·15H_2_O	Carcinogenic	[28,29,30,38]
Clinoptilolite	(Na,K,Ca)_2-3_Al_3_(Al,Si)_2_Si_13_O_36_·12H_2_O	Environmental purification, intestinal detoxifier, antioxidant, anti-inflammatory, antitumor	[39,40,41,42,43]
